# Young Cervical Cancer Patients May Be More Responsive than Older Patients to Neoadjuvant Chemotherapy Followed by Radical Surgery

**DOI:** 10.1371/journal.pone.0149534

**Published:** 2016-02-22

**Authors:** Jin Zhou, Xiong Li, Kecheng Huang, Yao Jia, Fangxu Tang, Haiying Sun, Yuan Zhang, Qinghua Zhang, Ding Ma, Shuang Li

**Affiliations:** 1 Department of Obstetrics and Gynecology, Tongji Hospital, Tongji Medical College, Huazhong University of Science and Technology, Wuhan, P.R. China; 2 Cancer Center, Renmin Hospital of Wuhan University, Wuhan, Hubei, P.R. China; 3 Department of Gynecology and Obstetrics, The Central Hospital of Wuhan, Wuhan, China; 4 Department of Obstetrics and Gynecology, Union Hospital, Tongji Medical College, Huazhong University of Science and Technology, Wuhan, P.R. China; The First Affiliated Hospital with Nanjing Medical University, CHINA

## Abstract

**Objective:**

To evaluate the effects of age and the clinical response to neoadjuvant chemotherapy (NACT) in patients with cervical cancer who received neoadjuvant chemotherapy followed by radical surgery.

**Methods:**

A total of 1,014 patients with advanced cervical cancer who received NACT followed by radical surgery were retrospectively selected. Patients were divided into young (aged ≤35 years, n = 177) and older (aged >35 years, n = 837) groups. We compared the short-term responses and survival rates between the groups. The five-year disease-free survival (DFS) and overall survival (OS) rates were stratified by age, NACT response, and FIGO stage.

**Results:**

The overall response rate was 86.8% in the young group and 80.9% in the older group. The young patients had an earlier FIGO stage (*P<*0.001), a higher rate of adenocarcinoma (*P* = 0.022), and more lymph node metastasis (*P* = 0.033) than the older patients. The presence of adenocarcinoma as the histological type (*P* = 0.024) and positive lymph node metastasis (*P*<0.001) were identified as independent risk factors for survival. When stratified by age and clinical response, young patients with no response to NACT had a worse clinicopathological condition compared with the other subgroups. Compared with non-responders, responders to NACT had a higher five-year DFS rate (80.1% versus 71.8%; *P* = 0.019) and OS rate (82.6% versus 71.8%; *P* = 0.003) among the young patients but not among the older patients.

**Conclusions:**

Responders to NACT aged 35 years or younger benefitted the most from NACT, while the young non-responders benefitted the least. Age might represent an important factor to consider when performing NACT in patients with cervical cancer.

## Introduction

Cervical cancer is the fourth most common cancer in women and one of the most common causes of cancer-related death in women worldwide [1]. Over the past four decades, with the widespread use of cervical cancer mass screening programs, the incidence and mortality rates of cervical cancer have declined. However, cervical cancer in young women remains common in China [2]. Patients younger than 35 years of age comprise approximately 16% of all patients with cervical cancer in China at present [3]. According to many earlier reports, a cervical cancer diagnosis at a young age is associated with more aggressive biological characteristics of the tumor and a more unfavorable prognosis compared with patients in whom the disease arises later [4–6]. The treatments used for cervical cancer often affect young women of reproductive age more severely than older patients due to the decrease in the quality of their sex life and the loss of future fertility. To be effective, treatment protocols must account for this age-related diversity in outcomes.

Radical surgery and radiotherapy have been the most common therapies for cervical cancer patients for decades [7]. However, radiation can lead to physiological dysfunction and a decreased quality of life. Adjunctive chemotherapy before and after surgery has been investigated with increasing attention over the past few years. More recently, neoadjuvant chemotherapy (NACT) followed by radical surgery (RS) has been gaining wider acceptance as an alternative treatment option [8–11]. According to several studies, NACT increases the chance of an optimal debulking surgery by shrinking the tumor(s) before surgery [12–14]. NACT can also reduce the risk of disease recurrence and death in patients without affecting reproductive functions while avoiding radiation-related complications [15]. In a phase III trial focused on NACT and radical hysterectomy versus radiotherapy for bulky early-stage cervical cancer, these two treatment modalities showed similar efficacies for bulky stage IB or IIA cervical cancer [10]. Therefore, based on these findings, NACT might represent a better treatment choice for young patients with locally advanced cervical carcinoma in order to preserve ovarian function. However, very few studies have compared details of the response to neoadjuvant chemotherapy between young and older patients, and no prospective study has focused solely on the outcomes of neoadjuvant chemotherapy in young patients [16]. As a result, whether young patients (≤35 years) with NACT have better outcomes than their older (**>**35 years) counterparts in terms of efficacy, tolerance, and survival following this treatment remains controversial.

In this study, we compared the clinicopathological factors and clinical responses to neoadjuvant chemotherapy between young and older cervical cancer patients. Simultaneously, we compared the survival rates among different subgroups divided according to age and response to neoadjuvant chemotherapy. We aimed to analyze the relationship between patient age and the response to neoadjuvant chemotherapy and whether patients in different age groups experience different levels of benefit from neoadjuvant chemotherapy.

## Patients and Methods

### Patients

All patients were retrospectively selected from the cervical cancer database v1.10 (http://clinicaltrials.gov; NCT01267851), which includes a total of 10,897 patients. This study was approved by the Ethics Committee of Huazhong University of Science and Technology, and all the participants provided their written consent to participate in this study.

The eligibility criteria were as follows: patients with squamous cell or adenosquamous carcinoma or adenocarcinoma of the cervix; patients with stage IB1–IIB disease according to the Federation of Gynecology and Obstetrics (FIGO) [17]; patients treated with NACT followed by radical hysterectomy; patients who did not receive primary radiotherapy or other treatment; and patients without renal, pulmonary, hepatic, bone marrow, or cardiac dysfunction.

A total of 1,014 patients were selected for this study (S1 Fig). All of these patients were treated between January 2002 and December 2008. For the analysis, we divided the patients into a young group (age ≤35) and an older group (age >35) [18, 19]. There were 177 women in the young group and 837 in the older group.

### NACT

Most of the patients in this study received platinum-based neoadjuvant chemotherapy. Generally, patients with early-stage disease and large tumor size (>4 cm) were considered to receive neoadjuvant chemotherapy. Some patients with FIGO stage IIB disease (tumor size >2 cm) could be treated with neoadjuvant chemotherapy followed by radical hysterectomy. The other patients treated with neoadjuvant chemotherapy were selected based on the doctors’ comprehensive judgment. The NACT regimens used in our study are summarized in S1 Table. The treatment was repeated at three-week intervals for a total of one to three cycles, based on patient tolerance and response. Several patients with a good response received additional courses of treatment.

### Monitoring the tumor response

The response to NACT was evaluated by comparing the tumor size at the initial diagnosis and after neoadjuvant chemotherapy. The tumor diameter was evaluated by a comprehensive combination of magnetic resonance imaging, computed tomography, B-ultrasonography results, and gynecologic examination. We multiplied the longest diameter by the greatest perpendicular diameter to obtain the approximate surface area for a single tumor; we also added the products of the diameters of all measured lesions if multiple lesions were found in a single organ. The response to chemotherapy was defined as a complete response (CR), a partial response (PR), stable disease (SD), or progressive disease (PD) according to the World Health Organization (WHO) criteria [20], where CR indicates a complete disappearance of all clinically detectable disease, PR indicates a 50% or more decrease in tumor size, SD indicates less than a 50% reduction in tumor size, and PD indicates an increase in tumor volume or the appearance of new lesions.

### Postoperative treatment

In our study, the postoperative therapy was not specified. Patients were followed up and treated with radiotherapy or chemotherapy according to the criteria of each hospital.

### Follow-up study

After the completion of treatment, patients were regularly followed-up every 3 months during the first year and then every 6 months thereafter. A small proportion of patients who were lost to follow-up were included in the survival data.

### Statistical processing

A chi-square test, an independent t-test, and Fischer’s exact test were used to compare various clinicopathological factors among the groups. Disease-free survival (DFS) and overall survival (OS) were estimated by Kaplan–Meier analysis. A log-rank test was used to compare survival curves. A value of *P*<0.05 was considered to be statistically significant. All of the statistical analyses were performed using SPSS software, version 13.0 (SPSS, Inc., Chicago, IL).

## Results

### 1. Patient characteristics

Baseline characteristics of the 1,014 patients in our study are provided in Table 1. Patient age ranged from 22 to 69 years, with a median age of 43 years. In total, 177 patients (17.5%) were aged 35 years or younger. Compared with patients over 35 years old, the young patients (≤35 years) had a lower proportion of advanced (IIA-IIB) FIGO stage disease (46.3% versus 61.6%, *P<*0.001), a higher rate of adenosquamous carcinoma or adenocarcinoma histology (15.3% versus 9.4%, *P* = 0.022), and a higher rate of lymph node metastasis (28.4% versus 20.8%, *P* = 0.033). However, no significant differences in differentiation, tumor size, NACT cycles, parametrial invasion, deep stromal invasion, or lymphovascular space invasion were found between the young and older patients.

**Table 1:** Comparison of the clinicopathological factors between young and older patients.

		Age (years)		
	No.	≤35	>35	*P*
Patient number	1014	177 (17.5%)	837 (82.5%)	
FIGO stage				
IB1	154	40 (22.6%)	114 (13.6%)	<0.001^a^
IB2	262	55 (31.1%)	207 (24.7%)	
IIA	275	37 (20.9%)	238 (28.4%)	
IIB	323	45 (25.4%)	278 (33.2%)	
IB1-IB2	416	95 (53.7%)	321 (38.4%)	<0.001
IIA-IIB	598	82 (46.3%)	516 (61.6%)	
Histological types				
Squamous cell carcinoma	908	150 (84.7%)	758 (90.6%)	0.022
Adenocarcinoma^b^	106	27 (15.3%)	79 (9.4%)	
Differentiation degree				
Low grade	208	37 (21.6%)	171 (21.3%)	0.927
High + Intermediate grade	765	134 (78.4%)	631 (78.7%)	
Tumor size				
≤4 cm	396	73 (41.2%)	323 (38.6%)	0.511
>4 cm	618	104 (58.8%)	514 (61.4%)	
Parametrial invasion				
Negative	922	160 (90.4%)	762 (91.0%)	0.786
Positive	92	17 (9.6%)	75 (9.0%)	
Lymphovascular space invasion				
Negative	914	158 (89.3%)	756 (90.3%)	0.668
Positive	100	19 (10.7%)	81 (9.7%)	
Lymph node metastases				
Negative	779	126 (71.6%)	653 (78.0%)	0.033
Positive	224	50 (28.4%)	174 (20.8%)	
Deep stromal invasion				
Negative	638	111 (62.7%)	527 (63.0%)	0.950
Positive	376	66 (37.3%)	310 (37.0%)	
NACT cycles				
1	629	100 (61.3%)	529 (67.7%)	0.250^a^
2	290	59 (36.2%)	231 (29.6%)	
≥3	25	4 (2.5%)	21 (2.7%)	

^a^ *P* values were calculated with a linear-by-linear association chi-square test.

^b^ Adenocarcinoma and adenosquamous carcinoma were included.

NACT, neoadjuvant chemotherapy.

### 2. Prognostic factors analysed by Cox proportional hazard models

Cox proportional hazard models were used to identify the prognostic factors for survival. An adenosquamous carcinoma or adenocarcinoma histological type (*P* = 0.024) and lymph node metastases (*P*<0.001) were identified as independent risk factors for survival (Table 2), both of which were more frequently found in patients aged 35 years or younger than in patients over 35 (Table 1).

**Table 2:** Multivariate analysis by Cox proportional hazard models for all patients.

	Hazard ratio	95% CI	*P*
Histological types			
Squamous cell carcinoma	1.000		
Adenocarcinoma^a^	1.887	1.086–3.278	0.024
Lymph node metastases			
Negative	1.000		
Positive	2.789	1.842–4.225	<0.001
Age			
≤35	1.000		
>35	1.204	0.717–2.021	0.483

^a^ Adenocarcinoma and adenosquamous carcinoma were included.

### 3. Response to NACT

The response to NACT by age group is listed in Table 3. The overall response (CR + PR) rate of the young patients was higher than that of the older patients, although the difference was not significant (86.8% versus 80.9%, *P* = 0.070). Additionally, young patients were more sensitive to NACT than were older patients among patients with an earlier (IB1-IB2) FIGO stage (91.2% versus 82.8%, *P* = 0.050) and among patients with cervical squamous cell carcinoma (88.0% versus 80.8%, *P* = 0.041). The young group showed a significantly better clinical response than did the older group (93.2% versus 81.8%, *P* = 0.018) among patients with stage IB1-IB2 and squamous cell carcinoma, as shown in Table 4. However, no significant difference was found in the response to NACT between the young group and older group when stratified by the presence of lymph node metastasis (Table 3).

**Table 3:** Clinical response to NACT between the two age groups.

	Age				
	≤35		>35		
	No.	%	No.	%	*P*^a^
Total					
CR	17	10.2	77	9.9	
PR	128	76.6	553	71	
CR+PR	145	86.8	630	80.9	0.070^b^
SD+PD	22	13.2	149	19.1	
FIGO stage					
IB1-IB2					
CR+PR	83	91.2	250	82.8	0.050
SD+PD	8	8.8	52	17.2	
IIA-IIB					
CR+PR	62	81.6	380	79.7	0.699
SD+PD	14	18.4	97	20.3	
Histological types					
Squamous cell carcinoma					
CR+PR	125	88.0	573	80.8	0.041
SD+PD	17	12.0	136	19.2	
Adenocarcinoma^c^					
CR+PR	20	80.0	57	81.4	0.876
SD+PD	5	20.0	13	18.6	
Lymph node metastases					
Negative					
CR+PR	106	88.3	515	83.7	0.204
SD+PD	14	11.6	100	16.3	
Positive					
CR+PR	39	83.0	115	70.1	0.080
SD+PD	8	17.0	49	29.9	

CR, complete response; PR, partial response; SD, stable disease; PD, progressive disease.

^a^ P value was calculated using the chi-square test.

^b^ P value was calculated by comparing the CR+PR group and the SD+PD group.

^c^ Adenocarcinoma and adenosquamous carcinoma were included.

**Table 4:** Clinical response to NACT between the two age groups in patients with stage IB1-IB2 and squamous carcinoma.

	IB1-IB2		
	≤35	>35	*P*^a^
Squamous cell carcinoma			
CR+PR	68 (93.2%)	220 (81.8%)	0.018
SD+PD	5 (6.8%)	49 (18.2%)	

NACT, neoadjuvant chemotherapy; CR, complete response; PR, partial response; SD, stable disease; PD, progressive disease.

^a^ P value was calculated using the chi-square test.

### 4. Survival analysis

Follow-up information was available for 807 (79.6%) of the 1014 total patients. The median duration of follow-up was 38 months (range: 2–110 months). Comparable five-year OS rates (79.9% versus 78.3%; *P* = 0.791) and DFS rates (77.9% versus 74.0%; *P* = 0.867) were found between the young and older groups (S2A and S2B Fig). Compared with the clinical non-responders, the clinical responders showed an increased DFS rate (*P* = 0.011; S2D Fig) and an improved OS rate (*P* = 0.065; S2C Fig), although the difference in OS was not significant.

**Fig 1:**
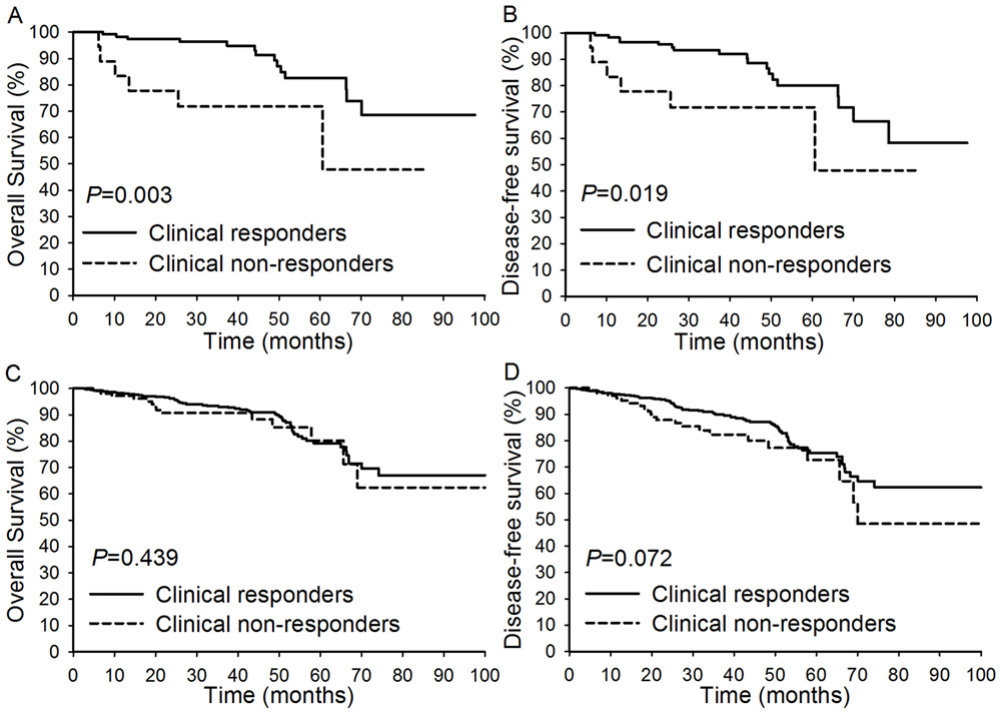
Overall survival and disease-free survival compared between clinical responders and clinical non-responders in different age groups. (A-B): The comparison of OS and DFS between clinical responders and clinical non-responders among patients aged 35 years or younger; (C-D): The comparison of OS and DFS between clinical responders and clinical non-responders among patients aged >35 years.

**Fig 2:**
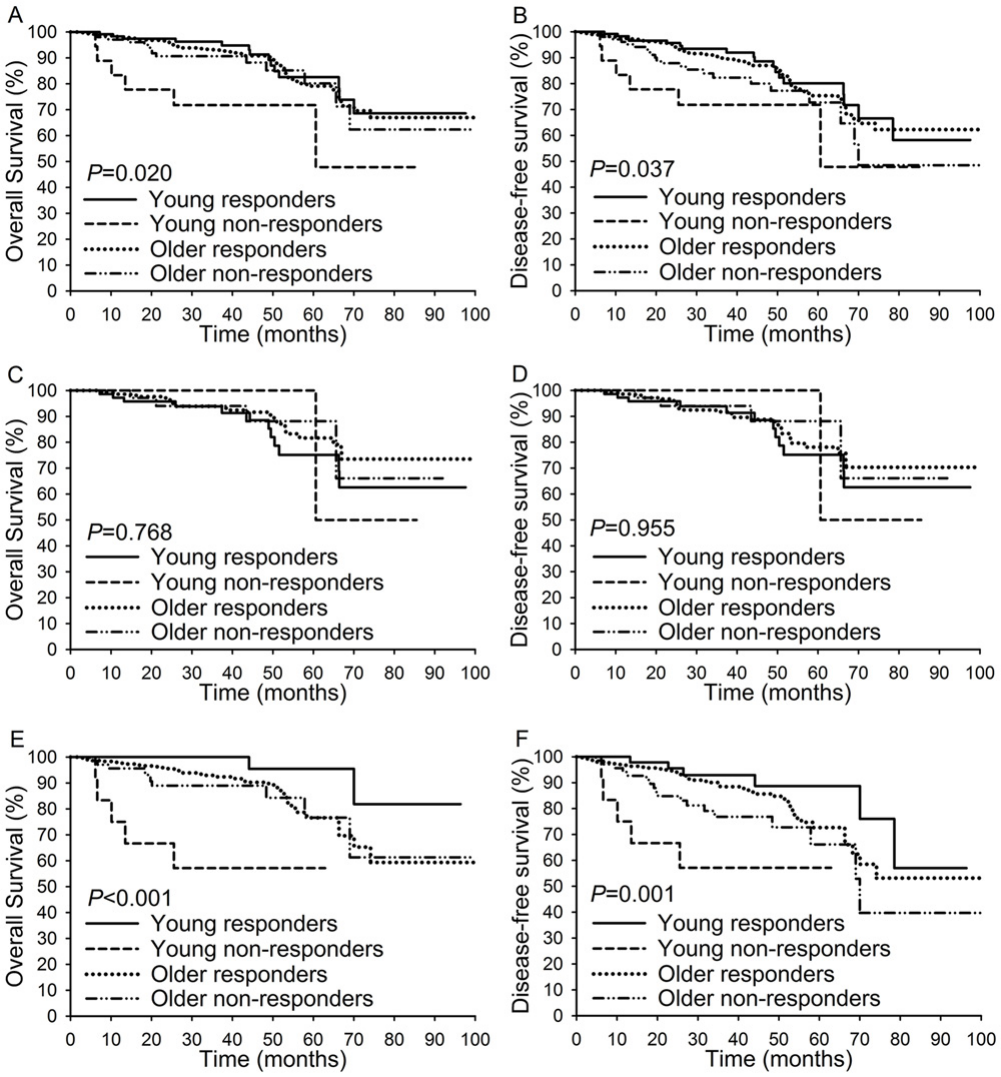
Overall survival and disease-free survival compared among patients of different subgroups. (A-B): The comparison of OS and DFS among the young responders subgroup, young non-responders subgroup, older responders subgroup, and older non-responders subgroup. (C-D): The comparison of OS and DFS among the young responders subgroup, young non-responders subgroup, older responders subgroup, and older non-responders subgroup among patients with FIGO stage IB1-IB2 disease. (E-F): The comparison of OS and DFS among the young responders subgroup, young non-responders subgroup, older responders subgroup, and older non-responders subgroup among patients with FIGO stage IIA-IIB disease.

Next, the clinical responders and clinical non-responders were stratified by age. As shown in Fig 1A and 1B, among patients aged 35 years or younger, the clinical responders had an increased five-year OS (82.6% versus 71.8%; *P* = 0.003) and five-year DFS (80.1% versus 71.8%; *P* = 0.019) compared with clinical non-responders. However, among patients over 35, the five-year OS rate (79.3% versus 80.2%; *P* = 0.439) and five-year DFS rate (75.5% versus 72.7%; *P* = 0.072) of the clinical responders were not significantly better than those of the clinical non-responders (Fig 1C and 1D). Compared with the other three subgroups, young non-responders showed the worst five-year OS rate (*P* = 0.020; Fig 2A) and the worst five-year DFS rate (*P* = 0.037; Fig 2B). When the clinicopathological factors were compared among the four subgroups (S2 Table), young non-responders showed the highest rate of adenocarcinoma (22.7%), lowest rate of differentiation (68.2%), highest rate of tumor size >4 cm (77.3%), highest rate of parametrial invasion (27.3%), highest rate of lymphovascular space invasion (59.1%), highest rate of lymph node metastasis (36.4%), and second highest rate of patients with an advanced FIGO stage (63.6%). When these four subgroups were stratified by FIGO stage, young non-responders showed the lowest five-year OS rate (*P*<0.001) and the lowest five-year DFS rate (*P* = 0.001) among patients with an advanced FIGO stage (Fig 2E and 2F) but not among patients with an early FIGO stage (Fig 2C and 2D). When we compared survival among the four subgroups in patients with squamous cell carcinoma and FIGO stage IIA-IIB disease, the DFS and OS rates of the young responders were higher than those of the other three subgroups, while the DFS (P<0.001) and OS (P<0.001) rates of the young non-responders were lower than those of the other three subgroups (S3 Fig).

## Discussion

Age is not clearly associated with the specific response to NACT in patients with cervical cancer. Although many investigators have assessed the association of age with the outcome of NACT patients [21], only a few studies have investigated patients ≤35 years of age [16]. Age thus remains a somewhat controversial topic with regard to its potential role as a predictive factor. The goal of this report was to evaluate the role of age and the efficacy of NACT in patients with cervical cancer.

Several studies have reported that compared with older patients, young patients had a higher proportion of non-SCC (squamous cell carcinoma) tumors and an earlier FIGO stage; they also showed a higher rate of lymph node metastasis than did older patients [5, 22]. Consistent with earlier reports, the present study also indicated that women aged 35 years or younger were significantly more likely than older patients to have adenocarcinoma. Adenocarcinoma generally tends to have earlier lymph node metastasis and to be less sensitive to chemotherapy than its squamous cell counterpart [23, 24]. Although younger age was not found to be an independent prognostic factor in the present study, the adenocarcinoma histological type and the presence of lymph node metastasis were found to be independent risk factors for poor survival (Table 2), both of which were associated with a younger age.

The overall response rate of young patients was slightly higher than that of older patients (Table 3). In a more in-depth analysis, our results clarified that in patients with stage IB1-IB2 disease and squamous cell carcinoma, the young patients had a better response than the older patients (Table 3). Bamias et al. reported that age was a significant predictor of the response rate when assessed as a continuous variable in a univariate model but was no longer considered an independent prognostic factor when assessed with other pretreatment variables in multivariate regression analysis [25]. In the current study, age remained a significant factor for the response rate within the earlier FIGO stage group and the squamous cell carcinoma group (Table 3). These findings suggest that young patients, especially those with earlier stage disease and the squamous cell histological type, tend to be more sensitive than older patients to NACT.

With regard to the survival rates, we did not find any significant differences between young and older patients. However, compared with non-responders, the five-year DFS and OS rates of responders were significantly increased among the young patients (Fig 1A and 1B), while the difference in the OS rates between responders and non-responders was not significant among the older patients (Fig 1C and 1D). When age and the response to NACT were considered simultaneously, the young responders had a significantly better OS rate compared with the other three subgroups, and the young non-responders had dramatically worse DFS and OS rates compared with the other three subgroups. The above findings were more significant among patients with an advanced FIGO stage (Fig 2E and 2F) but were not present among patients with an earlier FIGO stage. We also found that compared with the other three subgroups, young non-responders were more likely to have many poor clinicopathological factors. These results indicate that the response to NACT may affect young patients more strongly than older patients in terms of OS. OS was significantly prolonged by a favorable response to NACT in the young responders, while older responders still had poor outcomes.

The appropriate selection of patients who will benefit most from NACT is crucial. Our present findings indicate that young patients with an earlier FIGO stage and SCC showed a more favorable response than older patients. Young responders also had a better prognosis than young non-responders and older patients with stage IIA-IIB disease, while no difference was found between responders and non-responders among the older patients. These findings indicated that the OS of young patients was more strongly affected by NACT than was that of older patients. Our findings also suggested that NACT non-responders were unsuitable for NACT, especially among the young patients. However, no effective method is currently available to identify non-responders before NACT is performed. Additional studies are required to identify markers for the response to NACT. Future clinical studies should take age-related effects into consideration. Age-related effects may be helpful to predict which patients are likely to benefit from NACT and to avoid delaying the administration of effective treatment in patients who are unlikely to respond. Due to the limitations of retrospective studies, the neoadjuvant chemotherapy regimens before surgery and the treatments after surgery were varied. More detailed information that could be used to direct the choice of treatment regimens should be collected. Additional studies are required to validate the results of the present study.

## Supporting Information

S1 Fig Patient selection in the present study. (TIF)

S2 Fig Overall survival and disease-free survival compared between the young group and the older group. (A-B): The comparison of OS and DFS between the young group and the older group; (C-D): The comparison of OS and DFS between clinical responders and clinical non-responders.
(TIF)

S3 Fig Overall survival and disease-free survival among patients of different subgroups. (A-B): The comparison of OS and DFS among the young responders subgroup, young non-responders subgroup, older responders subgroup, and older non-responders subgroup among patients with squamous cell carcinoma and FIGO stage IIA-IIB disease.
(TIF)

S1 Table The chemotherapy regimen used in this study. (DOCX)

S2 Table Comparison of the clinicopathological factors among patients in subgroups divided by age and the clinical response to NACT. (DOCX)
