# Supplementary figures and images for: Young Cervical Cancer Patients May Be More Responsive than Older Patients to Neoadjuvant Chemotherapy Followed by Radical Surgery
Source: PLoS One. 2016 Feb 22;11(2):e0149534. doi: 10.1371/journal.pone.0149534 (PMC4763723; doi:10.1371/journal.pone.0149534)

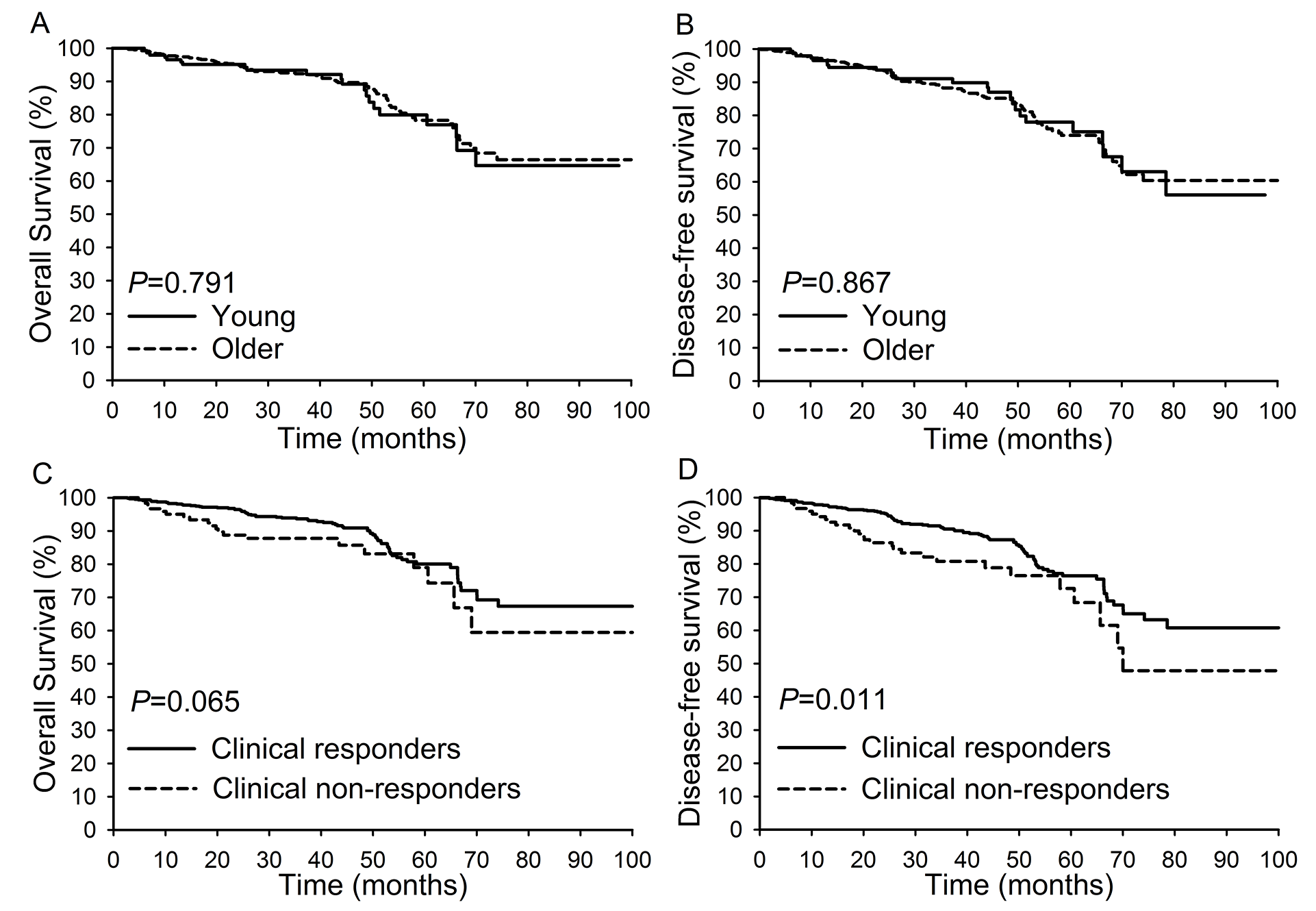

Supplement: S2 Fig — (A-B): The comparison of OS and DFS between the young group and the older group; (C-D): The comparison of OS and DFS between clinical responders and clinical non-responders. (TIF) [file pone.0149534.s002.TIF]

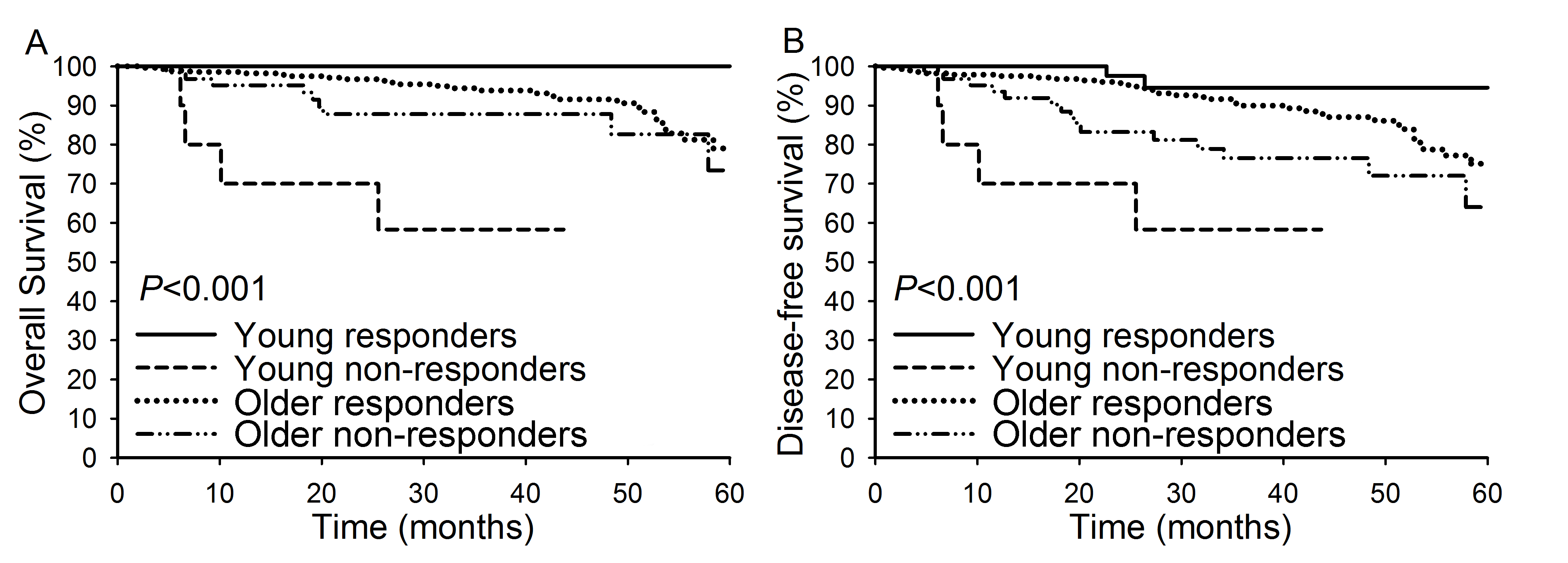

Supplement: S3 Fig — (A-B): The comparison of OS and DFS among the young responders subgroup, young non-responders subgroup, older responders subgroup, and older non-responders subgroup among patients with squamous cell carcinoma and FIGO stage IIA-IIB disease. (TIF) [file pone.0149534.s003.tif]
