# Supplementary material for: Young Cervical Cancer Patients May Be More Responsive than Older Patients to Neoadjuvant Chemotherapy Followed by Radical Surgery
Source: PLoS One. 2016 Feb 22;11(2):e0149534. doi: 10.1371/journal.pone.0149534 (PMC4763723; doi:10.1371/journal.pone.0149534)
Supplement: S1 Table — (DOCX) [file pone.0149534.s004.docx]

| **S1 Table.** The chemotherapy regimen used in this study. | | |
| --- | --- | --- |
| Schemes | Patients  Number and percent (%) | Chemotherapy regimens |
| TP | 372 (36.7%) | Paclitaxel 135-175 mg/m^2^ intravenously (IV) on day 1, and cisplatin 80-85 mg/m^2^ IV from days 2 to 3. This treatment was repeated every 28 days. |
| BVP | 165 (16.3%) | Blemycin 20 mg/m^2^/day IV on day 1 and day 3, VCR 1 mg/m^2^ IV on day 2, cisplatin 75 mg/m^2^ IV on day 1. Two cycles of NAC were initially given at 14-day intervals. |
| PF | 163 (16.1%) | Cisplatin 75 mg/m^2^ IV on day 1, and 5-Fu 24 mg/kg/d IV from day 1 to day 5. This treatment was repeated at 3-week intervals. |
| BP | 101 (10.0%) | Blemycin 20 mg/m^2^/day IV on day 1 and day 3, cisplatin 75 mg/m^2^ IV on day 1. Two cycles of NAC were initially given at 14-day intervals. |
| PFM | 73 (7.2%) | Cisplatin 100 mg/m^2^ intravenously IV on day 1, mitomycin C 4 mg/m^2^ intramusculary (IM) from day 1 to day 5, and 5-fluorouracil 24 mg/kg/day IV from Day 1 to Day 5. Two cycles of NAC were initially given at 14-day intervals. |
| CP | 46 (4.5%) | Irinotecan hydrochloride 60 mg/m^2^ IV on day 1, day 8, and day 15, and cisplatin 60 mg/m^2^ IV on day 1. This treatment was repeated every 28 days. |
| Others and unknown | 94(9.3%) |  |
