# Supplementary material for: Young Cervical Cancer Patients May Be More Responsive than Older Patients to Neoadjuvant Chemotherapy Followed by Radical Surgery
Source: PLoS One. 2016 Feb 22;11(2):e0149534. doi: 10.1371/journal.pone.0149534 (PMC4763723; doi:10.1371/journal.pone.0149534)
Supplement: S2 Table — (DOCX) [file pone.0149534.s005.docx]

**S2 Table.** Comparison of the clinico-pathological factors among patients in subgroups divided by age and the clinical response to NACT.

|  |  | ≤35 | |  | >35 | |  |
| --- | --- | --- | --- | --- | --- | --- | --- |
|  |  | CR + PR | SD +PD |  | CR + PR | SD +PD | *P* value^a^ |
| FIGO stage |  |  |  |  |  |  |  |
| IB1-IB2 |  | 83 (57.2%) | 8 (36.4%) |  | 250 (39.7%) | 52 (34.9%) | <0.001 |
| IIA-IIB |  | 62 (42.8%) | 14 (63.6%) |  | 379 (60.3%) | 97 (65.1%) |  |
| Histological types |  |  |  |  |  |  |  |
| Squamous |  | 125 (86.2%) | 17 (77.3%) |  | 572 (90.9%) | 136 (91.3%) | 0.068 |
| Adenocarcinoma^b^ |  | 20 (13.8%) | 5 (22.7%) |  | 57 (9.1%) | 13 (8.7%) |  |
| Differentiation degree |  |  |  |  |  |  |  |
| Low grade |  | 112 (78.9%) | 15 (68.2%) |  | 482 (79.0%) | 109 (75.2%) | 0.504 |
| High + Intermediate grade |  | 30 (21.1%) | 7 (31.8%) |  | 128 (21.0%) | 36 (24.8%) |  |
| Tumor size |  |  |  |  |  |  |  |
| ≤4 cm |  | 62 (42.8%) | 5 (22.7%) |  | 236 (37.5%) | 53 (35.6%) | 0.264 |
| >4 cm |  | 83 (57.2%) | 17 (77.3%) |  | 393 (62.5%) | 96 (64.4%) |  |
| Parametrial invasion |  |  |  |  |  |  |  |
| Negative |  | 135 (93.1%) | 16 (72.7%) |  | 582 (92.5%) | 129 (86.6%) | 0.002 |
| Positive |  | 10 (6.9%) | 6 (27.3%) |  | 47 (7.5%) | 20 (13.4%) |  |
| Lymphovascular space invasion |  |  |  |  |  |  |  |
| Negative |  | 98 (67.6%) | 9 (40.9%) |  | 418 (66.5%) | 65 (43.6%) | <0.001 |
| Positive |  | 47 (32.4%) | 13 (59.1%) |  | 211 (33.5%) | 84 (56.4%) |  |
| Lymph node metastases |  |  |  |  |  |  |  |
| Negative |  | 106 (73.1%) | 14 (63.6%) |  | 515 (81.9%) | 100 (67.1%) | <0.001 |
| Positive |  | 39 (26.9%) | 8 (36.4%) |  | 114 (18.1%) | 49 (32.9%) |  |

NACT, neoadjuvant chemotherapy; CR, complete response; PR, partial response; SD, stable disease; PD, progressive disease.

^a^ *P* values were calculated with R*C chi-square test.

^b^ Adenocarcinoma and adenosquamous carcinoma were included.
